# Supplementary material for: Prevalence and factors associated with Trichomonas vaginalis infection in indigenous Brazilian women
Source: PLoS One. 2020 Oct 16;15(10):e0240323. doi: 10.1371/journal.pone.0240323 (PMC7567381; doi:10.1371/journal.pone.0240323)
Supplement: S1 Checklist — (PDF) [file pone.0240323.s001.pdf]

STROBE Statement—Checklist of items that should be included in reports of ***cross-sectional studies***

	Item No	Recommendation	Reported on page #
**Title and abstract**	1	(*a*) Indicate the study's design with a commonly used term in the title or the abstract	P1L1/P2L26
		(*b*) Provide in the abstract an informative and balanced summary of what was done and what was found	P2L26-35
**Introduction**			
Background/rationale	2	Explain the scientific background and rationale for the investigation being reported	P3L42-50
Objectives	3	State specific objectives, including any prespecified hypotheses	P3L51-68
**Methods**			
Study design	4	Present key elements of study design early in the paper	P4L71-77
Setting	5	Describe the setting, locations, and relevant dates, including periods of recruitment, exposure, follow-up, and data collection	P4L79-85
Participants	6	(*a*) Give the eligibility criteria, and the sources and methods of selection of participants	P4L79-82
Variables	7	Clearly define all outcomes, exposures, predictors, potential confounders, and effect modifiers. Give diagnostic criteria, if applicable	P4L82-85
Data sources/ measurement	8*	For each variable of interest, give sources of data and details of methods of assessment (measurement). Describe comparability of assessment methods if there is more than one group	*82-86* *Table 2*
Bias	9	Describe any efforts to address potential sources of bias	*87-90*
Study size	10	Explain how the study size was arrived at	P4l75-77
Quantitative variables	11	Explain how quantitative variables were handled in the analyses. If applicable, describe which groupings were chosen and why	85-86 Table 2
Statistical methods	12	(*a*) Describe all statistical methods, including those used to control for confounding	129-139
		(*b*) Describe any methods used to examine subgroups and interactions	129-139
		(*c*) Explain how missing data were addressed	90-91
		(*d*) If applicable, describe analytical methods taking account of sampling strategy	129-139
		(*e*) Describe any sensitivity analyses	Not applicable
**Results**			
Participants	13*	(a) Report numbers of individuals at each stage of study—eg numbers potentially eligible, examined for eligibility, confirmed eligible, included in the study, completing follow-up, and analysed	P6L132-133
		(b) Give reasons for non-participation at each stage	P6L132/133
		(c) Consider use of a flow diagram	FIG 1
Descriptive data	14*	(a) Give characteristics of study participants (eg demographic, clinical, social) and information on exposures and potential confounders	P6L133-137
		(b) Indicate number of participants with missing data for each variable of interest	Not applicable
Outcome data	15*	Report numbers of outcome events or summary measures	Not applicable
Main results	16	(*a*) Give unadjusted estimates and, if applicable, confounder-adjusted	P6,7 and 8

		estimates and their precision (eg, 95% confidence interval). Make clear which confounders were adjusted for and why they were included	
		(*b*) Report category boundaries when continuous variables were categorized	Table 2
		(*c*) If relevant, consider translating estimates of relative risk into absolute risk for a meaningful time period	Not applicable
Other analyses	17	Report other analyses done—eg analyses of subgroups and interactions, and sensitivity analyses	Table 3
**Discussion**			
Key results	18	Summarise key results with reference to study objectives	P9L185-191
Limitations	19	Discuss limitations of the study, taking into account sources of potential bias or imprecision. Discuss both direction and magnitude of any potential bias	P11L237-242
Interpretation	20	Give a cautious overall interpretation of results considering objectives, limitations, multiplicity of analyses, results from similar studies, and other relevant evidence	P11L192-217/
Generalisability	21	Discuss the generalisability (external validity) of the study results	P11L218-236/ P11L243-247
**Other information**			
Funding	22	Give the source of funding and the role of the funders for the present study and, if applicable, for the original study on which the present article is based	P12L56-60

*Give information separately for exposed and unexposed groups.

Note: An Explanation and Elaboration article discusses each checklist item and gives methodological background and published examples of transparent reporting. The STROBE checklist is best used in conjunction with this article (freely available on the Web sites of PLoS Medicine at http://www.plosmedicine.org/, Annals of Internal Medicine at http://www.annals.org/, and Epidemiology at http://www.epidem.com/). Information on the STROBE Initiative is available at www.strobe-statement.org.
