# Supplementary material for: Prevalence and factors associated with Trichomonas vaginalis infection in indigenous Brazilian women
Source: PLoS One. 2020 Oct 16;15(10):e0240323. doi: 10.1371/journal.pone.0240323 (PMC7567381; doi:10.1371/journal.pone.0240323)
Supplement: Attachment — Submitted filename: Responses to Revielwers.docx [file pone.0240323.s003.docx]

**Revision Note**

September 17^th^, 2020

Dr. Catherine E Oldenburg
Academic Editor
PLOS ONE

Dear Editor

Thank you very much for inviting us to submit a revised version of our manuscript entitled: ***“*Prevalence and factors associated with Trichomonas vaginalis infection in indigenous Brazilian women (PONE-D-20-17319R1)*”*** for publication in **PLoS One**. We reviewed the points raised by reviewer 1 in this second review. Please find bellow a point-by-point response to the reviewer’s and to your comments.

We hope that the manuscript can be now reconsidered for publication in PLos One. Please, let me know if any further change in the manuscript is required.

Yours Sincerely,

**Silvana B. Marchioro (corresponding author)**

**DVM, PhD**

**Comments to the Author**

Reviewer #1: The article presented here is better structured and discussed. It is indicated for publication, according to my assessment. The indications made in the first review were corrected satisfactorily, and I don´t have any more major requests.
I have only two indications to do. The title of figure 1 (Fig 1. A sampling flowchart showing the number of samples collected and diagnostic methods performed) is found in the middle of the article (line 152-153). This must appear immediately before the figure, and not alone in the middle of the text.

#### R: We appreciate your observation. The title of the figure was inserted in the text following the journal rules in the guide for authors*. (Figure captions must be inserted in the text of the manuscript, immediately following the paragraph in which the figure is first cited (read order). Do not include captions as part of the figure files themselves or submit them in a separate document).*

The authors return the results data in the discussion, once again, the indication of the tables. I believe that this indication of the tables in the discussion is not necessary (lines 234 and 239), given that it was made in the results.

R: We excluded the indication of the tables and the p-values from the discussion as suggested by the reviewer. Thanks for watching this mistake and we apologize for not fixing it in the first time.

This is an article relevant to the field of public health and the data here exposed are well presented. I take this opportunity to thank you for the invitation and opportunity to review this work. Nothing more to say.

Kind regards,

F. S.

Reviewer #2: (No Response)
